# Preservation of Essential Odor-Guided Behaviors and Odor-Based Reversal Learning after Targeting Adult Brain Serotonin Synthesis

**DOI:** 10.1523/ENEURO.0257-16.2016

**Published:** 2016-11-17

**Authors:** Kaitlin S. Carlson, Meredith S. Whitney, Marie A. Gadziola, Evan S. Deneris, Daniel W. Wesson

**Affiliations:** Department of Neurosciences, Case Western Reserve University, Cleveland, OH, 44106

**Keywords:** 5-HT, odor discrimination, odor learning, operant behavior, psychophysics, reversal learning

## Abstract

The neurotransmitter serotonin (5-HT) is considered a powerful modulator of sensory system organization and function in a wide range of animals. The olfactory system is innervated by midbrain 5-HT neurons into both its primary and secondary odor-processing stages. Facilitated by this circuitry, 5-HT and its receptors modulate olfactory system function, including odor information input to the olfactory bulb. It is unknown, however, whether the olfactory system requires 5-HT for even its most basic behavioral functions. To address this question, we established a conditional genetic approach to specifically target adult brain *tryptophan hydroxylase 2* (*Tph2*), encoding the rate-limiting enzyme in brain 5-HT synthesis, and nearly eliminate 5-HT from the mouse forebrain. Using this novel model, we investigated the behavior of 5-HT–depleted mice during performance in an olfactory go/no-go task. Surprisingly, the near elimination of 5-HT from the forebrain, including the olfactory bulbs, had no detectable effect on the ability of mice to perform the odor-based task. *Tph2*-targeted mice not only were able to learn the task, but also had levels of odor acuity similar to those of control mice when performing coarse odor discrimination. Both groups of mice spent similar amounts of time sampling odors during decision-making. Furthermore, odor reversal learning was identical between 5-HT–depleted and control mice. These results suggest that 5-HT neurotransmission is not necessary for the most essential aspects of olfaction, including odor learning, discrimination, and certain forms of cognitive flexibility.

## Visual Abstract

**Figure:**
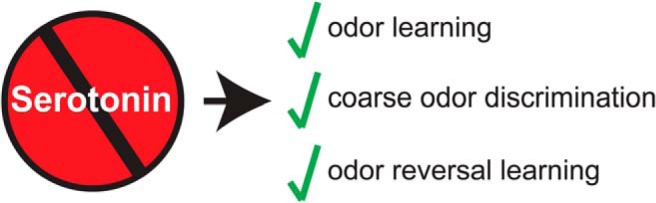

## Significance Statement

Modulation of sensory systems by neurotransmitters is considered critical for perception. The olfactory system is robustly innervated by serotonin (5-HT) neurons into both its primary and secondary odor-processing stages. Facilitated by this circuitry, 5-HT and its receptors modulate olfactory system function, including odor information input to the olfactory bulb. Here we asked whether the olfactory system needs 5-HT by using a conditional genetic approach to specifically target adult brain 5-HT synthesis and nearly eliminate 5-HT from the mouse forebrain. Our results suggest that 5-HT neurotransmission is not required for the most essential aspects of olfaction, including odor learning, odor discrimination, and odor-based cognitive flexibility. These findings raise questions about the importance and precise role of 5-HT modulation in olfactory system circuitry.

## Introduction

Our sensory systems must encode information under a wide range of dynamic contexts for survival. One way the brain handles this task is to modulate the activity within local sensory processing centers by means of extrinsic substances. In this manner, top-down centers that produce neuromodulators can differentially release these modulators in sensory centers depending on the needs of the animal. Major questions remain, however, regarding what neuromodulators are necessary for normal sensory system function (Katz, 1999; Hurley et al., 2004; Bouret and Sara, 2005; Linster and Fontanini, 2014).

The neurotransmitter serotonin (5-HT) is considered a powerful modulator of sensory system organization and function in a wide range of animals (Hen, 1992; Jacobs and Azmitia, 1992; Cases et al., 1996). The mammalian olfactory system is innervated by 5-HT neurons into both its primary and secondary odor processing stages (McLean and Shipley, 1987; Smith et al., 1993; Steinfeld et al., 2015; Suzuki et al., 2015). Midbrain 5-HT neurons located in the dorsal raphe nucleus (DRN) and median raphe nucleus (MRN) innervate the olfactory bulb, where fibers are observed in several cell layers (McLean and Shipley, 1987; Steinfeld et al., 2015; Suzuki et al., 2015; Muzerelle et al., 2016), including surrounding the glomeruli, which represent the first synaptic processing stage of odor information.

Facilitated by this circuitry, 5-HT and its receptors modulate olfactory system function (McLean et al., 1993; Aungst and Shipley, 2005; Hardy et al., 2005; Petzold et al., 2009; Liu et al., 2011a; Schmidt and Strowbridge, 2014; Brill et al., 2016; Brunert et al., 2016; Kapoor et al., 2016; Linster and Cleland, 2016; Lottem et al., 2016). For instance, electrical stimulation of the raphe nuclei modulates the level of odor information input into the olfactory bulb (Petzold et al., 2009). Additionally, optogenetic stimulation of raphe nuclei 5-HT neurons alters the representation of odors in several major populations of olfactory bulb neurons, including the primary output neurons (Brunert et al., 2016). Importantly, most of the above studies performed manipulations aimed at enhancing levels of synaptic 5-HT in the olfactory system. It is unknown, however, whether the olfactory system requires 5-HT for even its most basic behavioral functions.

In the present study, we investigated the behavior of mice with conditional depletions of adult brain 5-HT synthesis during their performance in an olfactory go/no-go task (Bodyak and Slotnick, 1999; Slotnick and Restrepo, 2001). To accomplish this, we used a recently established conditional approach (Whitney et al., 2016) to specifically target adult brain 5-HT synthesis, which nearly eliminates 5-HT from the entire mouse forebrain. Conditional targeting of adult brain 5-HT synthesis, together with well-established operant methods to robustly assay olfactory psychophysics, allowed to us test whether the adult olfactory system requires 5-HT for fundamental aspects of odor-guided operant behaviors, including odor learning and coarse odor discrimination. Our results suggest that adult brain 5-HT is not necessary for elementary function of the mammalian olfactory system.

## Materials and Methods

### Group design

**Figure 1.**
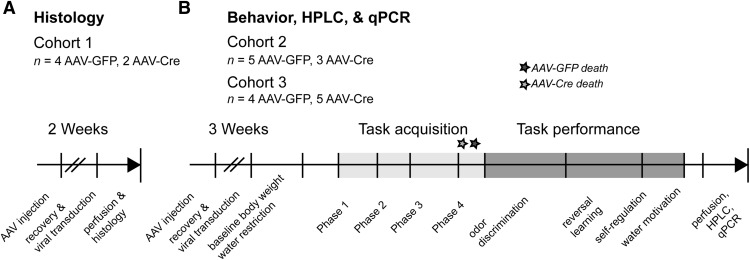
Experimental timeline for all mouse cohorts. Three separate cohorts of *Tph2^fl/fl^* male mice were used. ***A***, Cohort 1 was used solely for immunohistochemistry. ***B***, Cohorts 2 and 3 were used for olfactory go/no-go behavioral experiments and, after behavioral data collection, perfused for subsequent qPCR and HPLC analyses of postmortem brain tissue (see Materials and Methods). Two mice from the behavioral group (cohorts 2 and 3) were euthanized or died during the late stages of task acquisition after displaying signs of illness (indicated by stars).

Three different cohorts of *Tph2^fl/fl^* male mice (Kim et al., 2014) were used throughout this study (Fig. 1). These cohorts provided opportunities to confirm the depletion of adult 5-HT using immunohistochemical, molecular, and chemical methods as well as to test the functional effects of 5-HT depletion using behavioral methods.

### Surgical procedures and animal care

All animal procedures were in accordance with the guidelines of the National Institutes of Health and were approved by the Institutional Animal Care and Use Committee at Case Western Reserve University. Young adult mice (∼8 weeks of age) underwent a single survival intracranial surgical procedure to receive adeno-associated virus (AAV) as described in Whitney et al. (2016). After induction in isoflurane anesthetic (3.0–3.5% in 1 l/min O_2_), the mice were mounted into a stereotaxic frame and the anesthetic state was maintained under isoflurane. Core body temperature was maintained at 38°C with a heating pad. Upon confirmation of anesthesia depth, the head was shaved and cleaned with betadine and 70% EtOH, and a single injection of marcaine (s.c.) was administered within the site of the future wound margin. A single midline incision was made from ∼3 mm posterior of the nose along the midline to ∼3 mm posterior of lambda. Two holes (∼1-mm diameter) were drilled ±0.4 mm from the midline of the skull (–4.15 mm from bregma). A 10-µl Gastight 1701 Hamilton syringe (30-gauge needle with a 13° bevel; Hamilton, Reno, NV) loaded with AAV (either AAV1.CMV.PI.Cre.rBG or AAV1.CMV.PI.EGFP.WPRE.bGH; Penn Vector Core, Philadelphia, PA) was lowered into the intended injection site (–4.0 mm ventral), and 1 µl of AAV was infused at a rate of 100 nl/min. After the first injection, the syringe was slowly raised out of the brain and the process was repeated at the second site. After the second injection, the craniotomies and skull were closed. Rimadyl (carprofen, 5 mg/kg s.c.; Pfizer, Groton, CT) was administered daily for 3 d after surgery. Food and water were available *ad libitum* except during behavioral recordings. All animals were returned to group housing the day of surgery on a 12:12-h (light:dark) schedule. Any mice exhibiting delayed recovery or signs of illness (lethargy, immobility, ungroomed fur) throughout any point of experimentation were immediately euthanized and not used for future data collection.

### Histology

**Figure 2.**
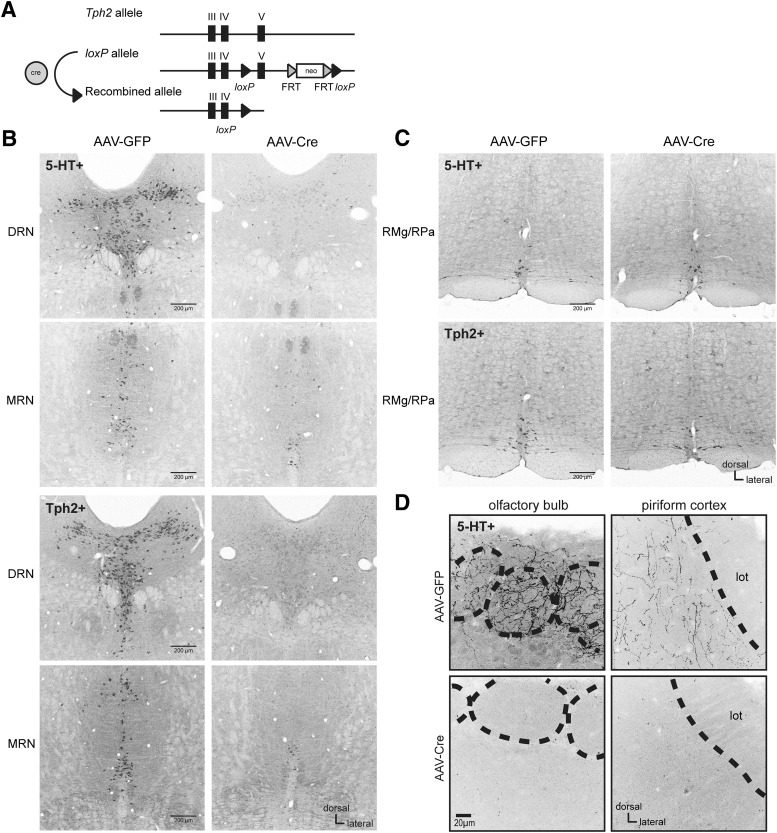
Targeting of *Tph2* in the brains of adult mice. ***A***, Schematic of wild-type *Tph2* allele (top), floxed allele with exon V flanked by *loxP* sites (middle), and targeted allele after Cre-mediated deletion of exon V (bottom). ***B***, Representative images of *Tph2^fl/fl^* mice confirming near-complete loss of 5-HT and Tph2 immunoreactivity within the MRN and DRN of AAV-Cre–treated mice. ***C***, Representative images of *Tph2^fl/fl^* mice illustrating the preservation of 5-HT– and Tph2-immunostained neurons in the medullary raphe, including the raphe magnus (RMg) and raphe pallidus (RPa) in AAV-Cre–treated mice. The raphe magnus does not send many fibers into the forebrain or olfactory structures specifically, but instead largely projects into the spinal cord (Bowker et al., 1981; Skagerberg and Björklund, 1985). ***D***, Representative images of *Tph2^fl/fl^* mice confirming near-complete loss of 5-HT immunoreactivity within both the main olfactory bulb and piriform cortex in AAV-Cre–treated mice. Images are from stacks of 1.2-µm-thick confocal images. Dashed lines in olfactory bulb images represent borders of glomeruli identified with nuclear counterstain (not shown), and in piriform lines, the border of the lateral olfactory track (lot) form layer i of the piriform. Images are from coronal sections, 20 µm thick. Images have been converted to monochrome and inverted. Contrast/brightness adjustments were equally applied to images in ***B*** and ***C*** to optimally display immunostained cell bodies. Contrast/brightness adjustments were equally applied to images in ***D*** to optimally display immunostained fibers.

As illustrated in Figure 1, a cohort of mice (cohort 1) not used in the behavioral testing was treated with AAV as described above and prepared for immunohistochemical staining for 5-HT and Tph2. Two weeks postsurgery, the mice were anesthetized with Avertin (0.5 g tribromoethanol/39.5 ml H_2_O, 0.02 ml/g) and perfused with cold PBS for 2–5 min, followed by cold 4% paraformaldehyde in PBS for 20 min. The brains were removed and cryoprotected in 30% sucrose:PBS overnight. Next, frozen coronal sections through the olfactory bulb and piriform cortex were obtained on a sliding microtome at 20-µm thickness and placed in 0.3% sodium azide/Tris-buffered saline at 4°C until staining. The remaining midbrain and hindbrain tissue was left in 30% sucrose:formalin until sectioning, when 20-µm frozen sections through all serotonergic nuclei were made on a sliding microtome. Similar sections from AAV-GFP– and AAV-Cre–injected mice were mounted on slides and vacuum-dried. They were then permeabilized in 0.3% Triton X-100:PBS (PBS-T) and blocked in 5% normal goat serum in PBS-T. Before blocking, antigen retrieval was performed only on slides containing sections through the serotonergic nuclei because of overfixation from formalin. Slides were placed in 10 mm sodium citrate, microwaved at low power for 10 min, cooled to room temperature, and washed three times for 5 min in PBS. All slides were then incubated in primary antibody in blocking solution O/N at 4°C, washed six times for 5 min in PBS-T, incubated in secondary antibody, and washed six times for 5 min in PBS-T. Coverslips were mounted with ProLong Gold antifade mountant with 4′,6-diamidino-2-phenylindole (Invitrogen, San Diego, CA). Primary antibodies used were rabbit anti-Tph2 (1:500; Millipore, Bedford, MA) and rabbit anti–5-HT (1:500; ImmunoStar, Hudson, WI), and the secondary antibody used was goat anti-rabbit Alexa 594 (Invitrogen). Coronal brain sections containing regions of interest were selected based on established boundaries (Paxinos and Franklin, 2000). Sections were imaged on a Zeiss LSM510 confocal microscope or a Zeiss Axioskop II MotPlus. Digital inversion of the images in grayscale and brightness/contrast adjustments were made in Adobe Photoshop equally for images within panels as noted (see Fig. 2).

### HPLC

Tissues from mice in cohorts 2 and 3 were prepared for HPLC analysis following established protocols (Lerch-Haner et al., 2008). Mice were anesthetized with Avertin and perfused with 10 U/ml heparin (Sigma-Aldrich, St. Louis, MO) in cold PBS for ∼6 min to clear the brain of blood and remove confounding peripheral 5-HT. Brains were immediately removed and placed on dry ice. When they were partially frozen, the brains were cut at bregma –2.92 mm to separate the forebrain and then again directly posterior to the olfactory bulbs. The forebrain and olfactory bulbs were immediately frozen on dry ice. Samples were shipped to the Neurochemistry Core of the Vanderbilt Brain Institute for processing and HPLC analysis of 5-HT and 5-hydroxyindoleacetic acid (5-HIAA) levels.

### Quantitative PCR

Mice from cohorts 2 and 3 were anesthetized with Avertin and perfused with 10 U/ml heparin (Sigma-Aldrich) in cold PBS for ∼6 min. Brains were immediately removed and placed on dry ice. When they were partially frozen, the brains were cut at bregma –2.92 mm and –5.68 mm to isolate midbrain tissue, which contains the DRN. With the section in a petri dish on a cold plate, a 1.5-mm tissue punch was made at midline, directly ventral to the third ventricle, to isolate the DRN. Tissue punches were lysed and homogenized using a 1-mL dounce homogenizer, from which RNA was isolated using a PureLink RNA Mini Kit (Invitrogen). RNA was quantified using a NanoDrop 2000 (Thermo Fisher Scientific, Waltham, MA). RNA (244 ng) from each sample was used for reverse transcription to cDNA with a Transcriptor First Strand cDNA Synthesis Kit (Roche, Basel, Switzerland). *Tph2* and *Actb* levels were quantified by quantitative PCR (qPCR) using TaqMan Fast Advanced Master Mix with TaqMan Gene Expression Assays for *Tph2* (Mm00557715_m1) and *Actb* (Mm00607939_s1; Applied Biosystems, Foster City, CA). The reactions were run in triplicate using a StepOnePlus system (Applied Biosystems), and relative expression values were calculated by StepOnePlus software with *Tph2* levels normalized to *β-actin* expression.

### Behavior

Mice used for olfactory go/no-go testing (cohorts 2 and 3; Fig. 1) were allowed 3 weeks to recover from surgery before any behavioral testing began. This duration also provided sufficient time for the AAV transduction and AAV-Cre–mediated targeting of brain *Tph2* (see Results). Baseline body weights were collected from all mice, and the mice were placed on a 24-h water restriction schedule with water available every 24 h in a small dish on their cage floor and/or in the context of behavioral task performance. We used a standard 80–85% body weight (from baseline weight) to ensure motivation in the operant water-motivated task (Slotnick and Restrepo, 2001). This level of weight loss is mild, and the mice appeared healthy (well-groomed fur, regular food intake) and active. After reaching 80–85% of baseline weight, mice were acclimated to the go/no-go operant boxes. Mice were single-housed for all behavioral procedures, and all testing occurred during the light phase of the cycle (0900 to 1800). All behavior was carried out in a dimly lit, well-ventilated room at 20–22°C.

**Figure 3.**
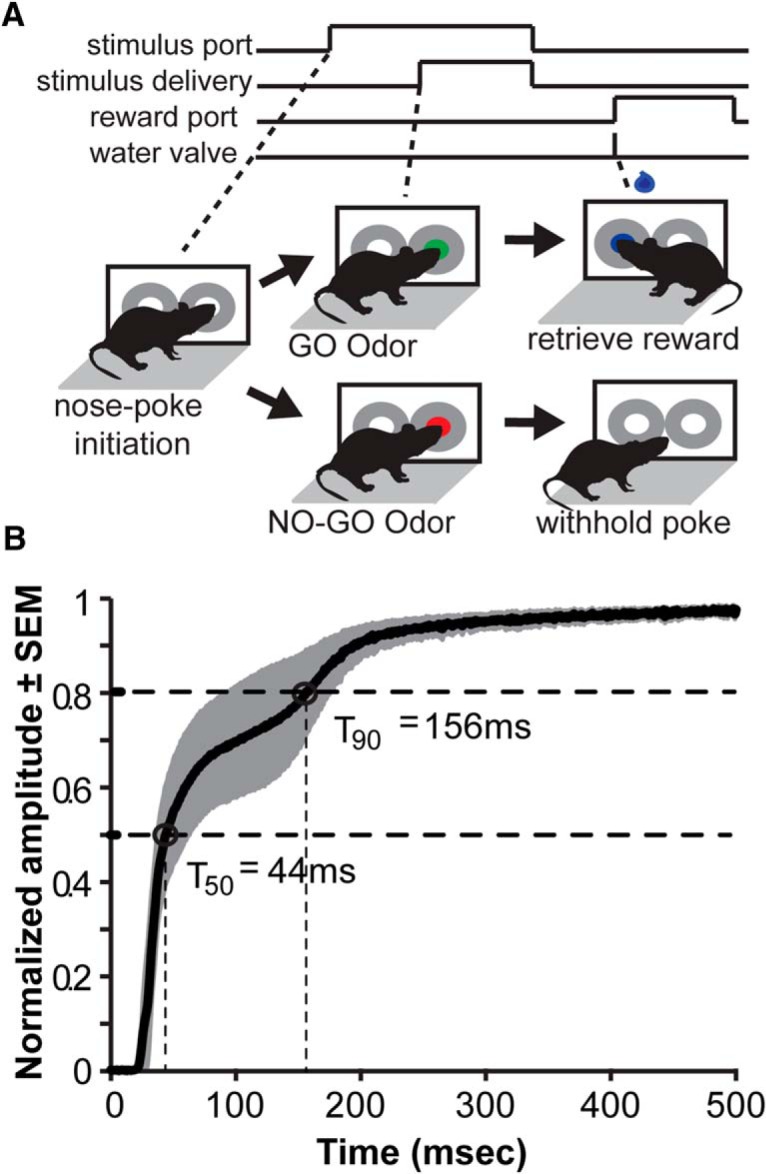
Go/no-go olfactory task design and stimulus control. ***A***, Outline of the go/no-go task structure as described in detail in Materials and Methods. In the final stage of the task, mice nose-poke in the right port (600-ms hold duration required), receive either CS+ or CS– (400-ms minimum hold [50-ms for self-regulation paradigm]; 2-s maximum hold), and retrieve a reward in the left port (S+ trial) or withhold their response (S– trial). Green shaded circle, presentation of CS+; red shaded circle, presentation of CS–; blue waterdrop icon, brief 3-µL water reward delivery. ***B***, Averaged voltage trace from a photoionization detector (PID) to illustrate the rapid odor stimulus dynamics as controlled by the go/no-go olfactometers. Fifteen trials of the odorant, heptanal (see Materials and Methods for intensity used and flow rate), were delivered by each olfactometer while the PID sampling port was positioned in the center of the odor sampling port. Data are normalized to the maximum value acquired by each olfactometer’s averaged PID output (over the 15 trials) and plotted as the average across all three olfactometers. Time 0 equals odorant valve onset. Time points of 50% (*T*_50_) and 90% rise times (*T*_90_) are indicated. Gray-shaded area indicates SEM. Although these dynamics may vary slightly across odors, this measure illustrates the precision and stability of the odor presentation methods used.

We used three custom-built go/no-go operant chambers designed based on the work of Bodyak and Slotnick (1999) and Slotnick and Restrepo (2001). The chambers were constructed of quarter-inch-thick acrylonitrile butadiene styrene (ABS) plastic and custom 3D-printed [polylactic acid (PLA)] nose-poke ports. The inner dimension of the chamber was 6 × 6 inches, with 11-inch-tall walls. On one wall was an operant plate consisting of two holes housing the two nose-poke ports (Fig. 3*A*). The nose-poke ports were three-quarter-inch inner diameter and were positioned 2 inches apart (center to center); each was 1.25 inches above the chamber floor (from floor to center of port). On another wall of the operant chamber was a hinged door allowing placement and removal of the mice. The chambers were open-top (no ceiling), to facilitate air circulation. The top of the odor port terminated into a flexible air hose (1-inch diameter) which was connected to a 12-V computer fan, which drew air from within the chamber (through the poke hole) up through the fan. An infrared LED and infrared photodetector were placed ∼2 mm into each port to provide continuous measures of port entry (beam interruption). The chambers were each housed in a single wooden enclosure box with an 8-inch-wide 12-V computer fan on one wall of the enclosure and a vent hole on the other to ensure that air could freely circulate throughout the chamber and facilitate odor elimination from within. All valves, computer hardware, and odor vials were housed outside of the enclosure boxes, which were positioned on a stainless steel rack.

Odor solenoid valves (Parker Hannifin, Cleveland, OH) and reward pinch valves (NResearch, West Caldwell, NJ) were controlled by custom code written in Tucker-Davis Technologies software (Alachua, FL), which gated voltage through a relay driver module (LabJack Corp., Lakewood, CO). The status of the infrared nose-poke beams as well as valve activity (odor or reward) was relayed into a digital processor (Tucker-Davis Technologies) and acquired to a computer at 3 kHz sampling rate.

Mice were shaped in the go/no-go task across four phases (Bodyak and Slotnick, 1999; Slotnick and Restrepo, 2001). In phase 1, upon nose-poke into the odor port (in absence of odor), the mice were allowed to withdraw and then nose-poke into the reward port in exchange for the water reward (∼3 µl). For the first two blocks of this, to facilitate task acquisition, the nose poke into the odor port automatically triggered water reward release, without the need for the animal to even poke within the reward port. Throughout phase 1, the duration required for the mouse to hold its nose in the port (break the beam) was gradually increased from 200 to 600 ms in 200-ms increments. Thus, by the end of phase 1, the mouse must be holding its nose in the odor port for 600 ms. Upon achieving ≥85% correct responses in two consecutive blocks of 20 trials (criterion performance), the mice were switched into phase 2 of training. In phase 2, the mice were required to nose-poke for 600 ms and then sustain the poke for an additional 200 ms, during which time a conditioned reward odor (CS+) was delivered (800 ms total hold requirement). The CS+ odor was delivered in all trials in which the animal held its nose in the port for ≥600 ms. The mice were then gradually required to increase their hold duration to remain with their noses in the port for 400 ms of CS+ odor presentation (1000 ms total hold requirement). Upon achieving criterion performance on phase 2, mice were transitioned to phase 3, wherein they had to detect the CS+ odor trials versus blank stimulus trials, in which reward delivery in the reward port occurred only upon nose pokes into this port preceded by CS+. During both trials, total hold duration was 1000 ms (600 ms, followed by 400-ms stimulus). Withdrawal from the odor port and poking in the reward port during CS+ trials resulted in a water reward. Upon achieving criterion performance on phase 3, mice were transitioned to phase 4 and shaped on the odor discrimination task. In this, the mice were again required to nose poke in the odor port for a minimum of 1000 ms (600 ms, followed by 400-ms stimulus). Withdrawal from the odor port and poking in the reward port during CS+ trials resulted in a water reward (hit). Delivery of CS+ but failure to poke in the reward port within 5 s was counted as an error (miss). Delivery of a conditioned nonrewarded odor (CS–) and failure to poke in the reward port within 5 s was counted as a correct reject. Finally, delivery of a CS– followed by poking in the reward port within 5 s was counted as a false alarm. The percentage of correct responses (hits and correct rejects vs. misses and false alarms) was determined for each block of 20 trials. During phase 4, a minimum 5-s intertrial interval was enforced. Mice were not cued to nose poke but instead were able to self-initiate trials by nose poking beyond the boundaries of the intertrial interval. The intertrial interval could thus be reset after completion of new trials or because of “short samples,” wherein the animal failed to maintain nose poke for the required duration. All mice contributing complete phase 4 data in the study were required to complete the same number of blocks (100 ± 5) before going onto the reversal learning task. During the reversal task, the behavioral contingencies for the CS+ and CS– were switched so that the previous CS– is now a CS+ (rewarded) and *vice versa*.

After reversal learning tests, the mice were required to perform in six blocks at or above criterion level (85%) before engagement in the odor-discrimination self-regulation task, wherein they were mandated to nose poke for only 600 ms and continue to nose poke for 50 ms of odor. In this overall testing structure, all mice needed to reach and demonstrate consistent criterion levels of behavior before being transitioned into the subsequent tasks.

Finally, after all olfactory testing, the mice were tested for water motivation on the next day. For water motivation tests, mice were placed in the operant chambers for 1 h, during which each nose poke into the odor port (in absence of odor) immediately triggered a reward to be released in the reward port on a fixed ratio 1 schedule.

The operant boxes were cleaned thoroughly with water and 90% EtOH between all behavioral sessions and mice and allowed to dry. Mice were tested only once per day.

### Stimulus presentation

Odors were presented through a custom air-dilution olfactometer with independent polytetrafluoroethylene (PTFE) stimulus lines up to the point of entry into the odor port. Odorants included ethyl butyrate, heptanal, isopentyl acetate, (-)-limonene (Sigma-Aldrich), each at their highest available purity (>97%) and each diluted to 0.5 Torr vapor pressure in mineral oil. These odors elicit distinct patterns of main olfactory bulb activity (e.g., Johnson et al., 2002), and thus they were selected for our assay of “coarse” olfactory discrimination. Odors and a blank stimulus (mineral oil) were presented at a rate of 1 l/min. Liquid odor (2 ml) was aliquoted into 25-ml glass headspace vials sealed with Teflon septa (Shamrock Glass, Seaford, DE), and air flow through the vials was permitted with 18-Ga s/s needles fit with PTFE lure fittings that terminated into PTFE odor lines (1/16-inch i.d.; Clippard Minimatic, Cincinnati, OH). Not all animals were tested with all odors. One cohort of mice used for behavior was initially shaped on one odor pair, whereas the other behavioral cohort was shaped on a different odor pair. The experimenter was not blind to odor assignment, but all stimulus presentation was automated. Rewarded and unrewarded odors were pseudorandomized within each block (10 trials of each CS+ and CS–). The concentrations of odorants were selected to be well above detection thresholds for mice (Bodyak and Slotnick, 1999). Odors were presented until the animal withdrew from the odor port or for a maximum duration of 2000 ms.

### Data analysis

Behavioral data acquired from the operant chambers were extracted in custom code written in Spike2 (Cambridge Electronic Design, Cambridge, UK). Data were extracted by a single experimenter before the unblinding of this experimenter to treatment groups. All statistical tests were performed in StatView (SAS Institute, Cary, NC). Data were pooled across cohorts within measures, organized by treatment group (AAV-GFP vs. AAV-Cre), and confirmed as normally distributed with a Kolmogorov–Smirnov test. Statistical *p* values are 2-tailed unpaired *t*-tests unless otherwise specified. Values are reported as mean ± SEM unless otherwise indicated.

## Results

### Conditional targeting of adult brain 5-HT synthesis

Three cohorts of mice were used to validate adult brain-specific *Tph2* targeting and test the necessity of adult brain 5-HT in odor-guided learning and olfactory perception (Fig. 1). We used a recently established protocol (Whitney et al., 2016) to specifically target adult brain 5-HT synthesis by stereotaxic injection of an AAV-Cre recombinase vector into mice (Kim et al., 2014) that have loxP sites flanking the fifth exon of *tryptophan hydroxylase 2* (*Tph2^fl/fl^*), which encodes the rate-limiting enzyme for the production of 5-HT (Fig. 2*A*). In adult *Tph2^fl/fl^* mice, AAV-Cre or AAV-GFP was injected into the midbrain, where 5-HT neurons of the MRN/DRN are located and project to olfactory structures in the forebrain (McLean and Shipley, 1987; Steinfeld et al., 2015).

In an independent cohort of *Tph2^fl/fl^* mice, we performed anti–5-HT and anti-Tph2 immunostaining to verify the targeting of *Tph2* and 5-HT depletion after injection of either AAV-Cre or AAV-GFP into the mice at 6 weeks of age (cohort 1, *n* = 2 and 4, respectively; Fig. 2). Analyses performed 2 weeks after injection of AAV-Cre into *Tph2^fl/fl^*mice indicated a near-complete loss of 5-HT and Tph2 immunoreactivity in the MRN and DRN, compared with AAV-GFP–injected mice (Fig. 2*B*). In contrast, and as an example of the precision of this approach, 5-HT and Tph2 immunoreactivity were preserved in the medullary raphe, which provide 5-HT innervation to the spinal cord (Bowker et al., 1981; Skagerberg and Björklund, 1985; Fig. 2*C*). Although virally mediated Cre expression is not restricted to 5-HT neurons, this targeting is specific to the 5-HT system, as *Tph2* is only expressed in 5-HT–producing neurons (Walther et al., 2003). Importantly, 5-HT–immunopositive fibers were strikingly absent in major olfactory structures, including in all the cell layers of main olfactory bulb and piriform cortex (Fig. 2*D*). Particularly in the olfactory bulb, the massive amounts of anti–5-HT fibers originating in the MRN and terminating in the glomerular layer (McLean and Shipley, 1987; Steinfeld et al., 2015; Suzuki et al., 2015; Muzerelle et al., 2016), the first synaptic processing layer of odor information, were absent in AAV-Cre–injected *Tph2^fl/fl^*mice (Fig. 2*D*). A rare and isolated 5-HT–immunopositive fiber was observed in occasional AAV-Cre–treated *Tph2^fl/fl^*mouse brain sections, including in the olfactory bulb granule cell layer. Although not shown here, 5-HT fibers were also absent in other olfactory structures, including the olfactory tubercle and anterior olfactory nucleus. Thus, as reported recently (Whitney et al., 2016), treatment of adult *Tph2^fl/fl^* mice with AAV-Cre achieves a near-complete loss of brain 5-HT, including, as shown here, in the olfactory bulb and piriform cortex.

### Learning and performance of adult *Tph2*-targeted mice in an olfactory go/no-go task

Having verified a conditional approach for the near-complete elimination of 5-HT in the adult forebrain including olfactory structures, we next sought to investigate our main hypothesis that adult brain 5-HT is necessary for essential odor-guided behaviors. There are countless assays available to explore olfactory perceptual function in mice. We selected the well-established, nose-poke–based olfactory go/no-go task (Bodyak and Slotnick, 1999; Slotnick and Restrepo, 2001) based on Pfaffmann et al. (1958) (Fig. 3). This operant task requires water-restricted mice to perform an instrumental water-motivated response to a conditioned odor; throughout conditioning, the behavior of the animal is motivated by thirst versus odor-specific motivation. This is important, since odor-specific motivation may alter odor investigatory dynamics (sniffing), a variable that may be compounded by 5-HT manipulations, which may modulate motivated behavior (Liu et al., 2014). In the go/no-go task, animals must learn to engage in the operant behavioral sequence of nose poking into an odor port to allow the possibility of receiving a 3-µl drop of water from the neighboring reward port. Reward delivery in the reward port occurs only upon nose pokes into this port preceded by the conditioned rewarded odor (CS+), but not the conditioned unrewarded odor (CS–; Fig. 3*A*). CS+ and CS– odor trials occurred in a pseudorandom order throughout all testing sessions.

Training in the go/no-go task occurs over four phases (see Materials and Methods), and thus a potential initial hurdle in our investigation into the olfactory behavior of 5-HT–depleted mice was shaping them to criterion performance (≥85% correct responses in two consecutive blocks of 20 trials). Indeed, 5-HT is considered a potent modulator of odor learning in neonatal rats (McLean et al., 1993). As before, 6-week-old adult *Tph2^fl/fl^* mice (cohorts 2 and 3) were injected with AAV-GFP or AAV-Cre into the midbrain DRN/MRN region. Mice were placed on a mild 24-h water restriction schedule at 23–29 d post-treatment to allow sufficient time for the near-complete elimination of brain 5-HT levels before shaping on the task started (see timeline in Fig. 1). One AAV-Cre–treated mouse was eliminated from all data analysis and statistical reports after both 5-HT HPLC and *Tph2* qPCR results that revealed intact brain 5-HT and *Tph2* levels, likely because of imprecise AAV injection.

**Figure 4.**
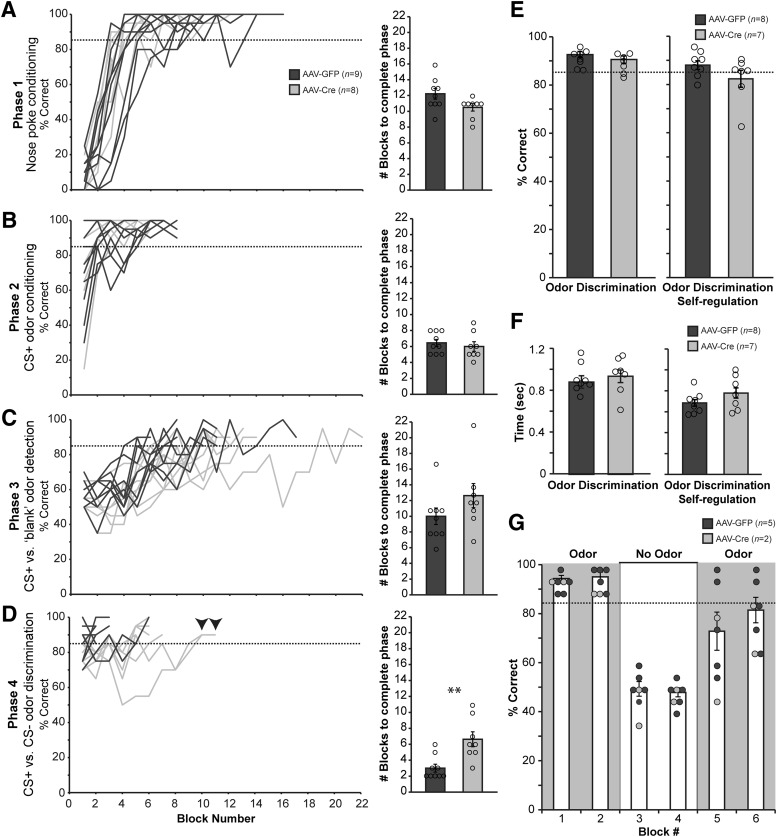
Adult *Tph2*-targeted mice learn the olfactory go/no-go task and display similar levels of odor acuity and odor sampling durations. ***A–D***, Learning curves (left) and total number of blocks required to complete each phase (right) across phases 1–4. **p < 0.005. ***E***, Average block percent correct performance during the odor discrimination task when odor sampling time was fixed (left, 2000 trials/mouse) and self-regulated (right, 300 trials/mouse). ***F***, Average sampling durations (nose poking during odor on) for CS+ and CS– odors during phase 4 odor discrimination (blocks ≥85%, 300 trials/mouse, left), and during self-regulation of odor discrimination (blocks ≥85%, 300 trials/mouse, right). ***G***, Odor-removal control experiment to demonstrate the reliance of the mice on the odor stimuli to engage in the go/no-go task. Data are mean ± SEM of all mice/block. Dots: individual mouse data.

Impressively, all remaining mice in the AAV-GFP (*n* = 9) and AAV-Cre (*n* = 8) treatment groups were able to learn the go/no-go task (Fig. 4). *Tph2*-targeted mice required a similar number of training blocks as control mice to learn reward retrieval in phase 1 (Fig. 4*A*; *t*(15) = –0.655, *p* = 0.522), sampling of the CS+ odor in phase 2 (Fig. 4*B*; *t*(15) = –0.028, *p* = 0.978), and odor detection of the CS+ odor from a blank stimulus in phase 3 (Fig. 4*C*; *t*(15) = –0.724, *p* = 0.480). When learning to discriminate the CS+ odor from a CS– odor in phase 4, *Tph2*-targeted mice required significantly more training blocks to reach performance criterion (Fig. 4*D*; *t*(15) = 3.507, *p* = 0.003). However, this finding was largely attributable to longer learning latencies in just two of the eight AAV-Cre mice, which eventually exceeded the criterion threshold (Fig. 4*D*, downward arrowheads).

With both groups of mice having learned the go/no-go task, we next allowed them to perform in the odor discrimination phase over multiple successive daily sessions (five to nine sessions) for a total of 2000 trials each (100 blocks/mouse). This total, 100 blocks of performance, was selected as a behavioral milestone to allow a large sampling of behavior from all animals and ensure extensive, as well as equivalent, task experience before engaging in the reversal learning paradigm. One AAV-Cre– and one AAV-GFP–treated mouse were eliminated from this and further data acquisition because of complications occurring within the 2 weeks of behavioral shaping, which impaired performance in the task (see Fig. 1). As shown in Fig. 4*E*, the remaining AAV-Cre–treated mice achieved similar behavioral performance as AAV-GFP–treated mice on the odor discrimination task, whether the mandatory odor sampling time was fixed at 400 ms (*t*(13) = –1.073, *p* = 0.306) or was self-regulated in a less restricted task structure (see Materials and Methods) performed across 15 blocks on subsequent sessions (*t*(13) = –1.486, *p* = 0.161). Similarly, over the course of the first 15 blocks of criterion performance, AAV-Cre mice sampled odors, measured as the time from odor onset to withdrawal from the sampling port, for similar durations compared to AAV-GFP mice (Fig. 4*F*), in both the original odor discrimination task (*t*(13) = 0.577, *p* = 0.574) and when odor sampling was self-regulated (*t*(13) = 1.358, *p* = 0.198). Thus, while both groups sampled odors for qualitatively less time in the self-regulated task compared to the original fixed odor sampling sessions (Fig. 4*F*, right vs. left), AAV-Cre mice maintained odor discrimination accuracy and odor sampling times similar to those of AAV-GFP mice on both task structures.

In a separate session, we sought to confirm that the mice were indeed relying on olfactory cues to perform the go/no-go task. In this experiment, a subset of mice (*n* = 7) shaped to criterion performance on the CS+ versus CS– phase 4 odor discrimination task were tested for their reliance on the odors to make correct responses. After two blocks of CS+ versus CS– odor discrimination (Fig. 4*G*, shaded), the experimenter disconnected the odor input lines to the odor ports of the operant chamber (Fig. 4*G*, unshaded). The reduced performance on block 3 and proceeding into block 4 falls within chance levels (<60% correct responses) in all mice. At block 5, the experimenter reconnected the odor lines, and the performance of the mice gradually returned (Fig. 4*G*, shaded). This illustrates that the mice were indeed using the odors to guide their behaviors, not other cues that may be associated with the operant chamber function (valve clicks, air flow, etc.).

### Gross motor control, water intake, body weight, and water motivation of *Tph2*-targeted mice

**Figure 5.**
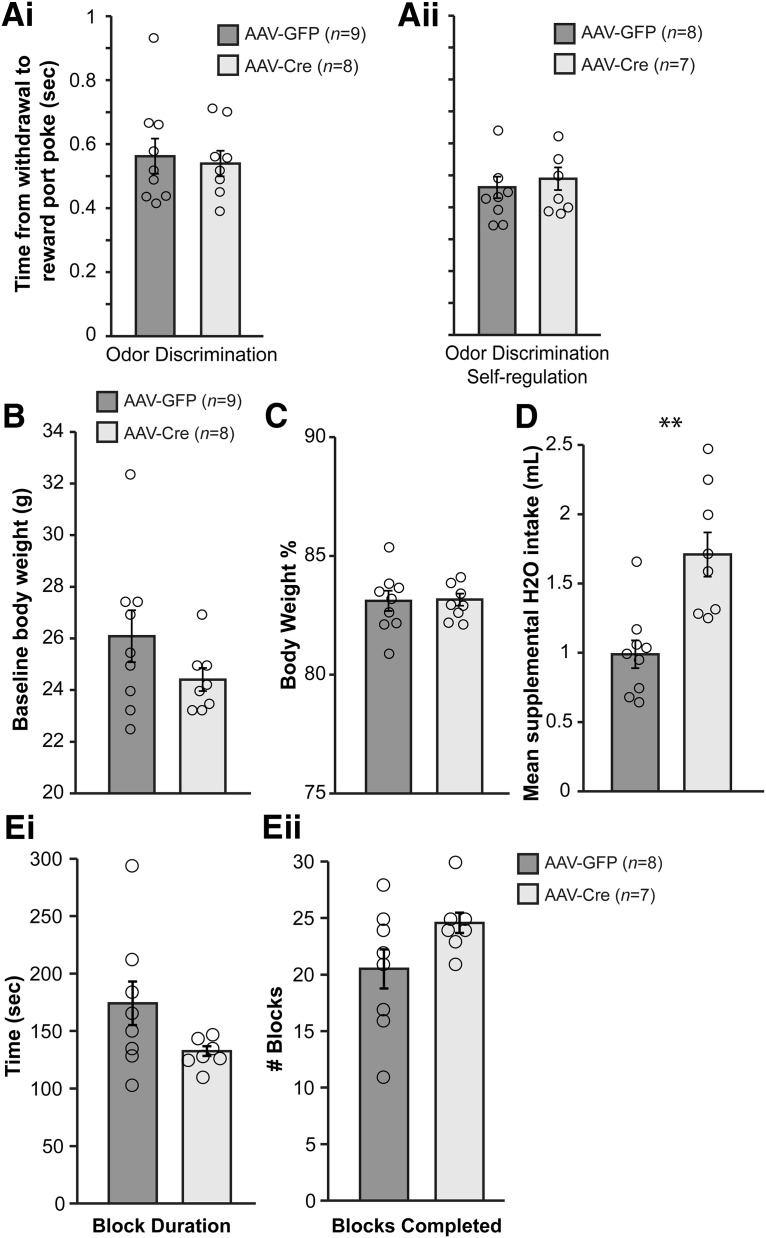
Gross motor performance, body weights, water intake, and water motivation of AAV-GFP– and AAV-Cre–treated *Tph2^fl/fl^* mice during the go/no-go task. ***A***, Average duration of withdrawal from odor port to nose poke in the reward port during phase 4 odor discrimination (15 blocks ≥85%, 150 CS+ trials/mouse; ***Ai***) and the self-regulation odor discrimination testing (15 blocks ≥85%, 150 CS+ trials/mouse; ***Aii***). ***B***, Baseline body weights of mice used in olfactory go/no-go testing before water deprivation. ***C***, Mean body weights of all mice (averaged across all days of behavioral testing, range 21–24 days) expressed as percentage of weight (during water restriction) as a function of baseline weight (***A***). ***D***, The mean of supplemental water for each mouse across all testing days. ***E***, Water motivation test results. Histograms of the average block duration (11–30 blocks/mouse; ***Ei***) and the number of blocks completed in a single 1-h session of the water motivation test (***Eii***). Data are mean ± SEM of all mice. Dots: individual mouse data. **p < 0.005.

To identify any additional factors that may influence go/no-go task performance in adult *Tph2-*targeted mice, we performed further measures on the mice (cohorts 2 and 3) either during task engagement or after completion of all olfactory testing. First, we measured gross motor control during task performance as assayed by the average duration of withdrawal from the odor port until nose poke in the reward port in phase 4 odor discrimination testing (Fig. 5*Ai*) and during the self-regulation odor discrimination testing (Fig. 5*Aii*). These data revealed that *Tph2*-targeted mice maintained coordinated performance (nose poking and movement between ports) in both phase 4 (*t*(15) = 1.809, *p* = 0.198) and the self-regulation testing (*t*(13) = 0.548, *p* = 0.593) compared to the AAV-GFP treated mice.

We also monitored body weights and fluid intake of the water-restricted mice. AAV-Cre– and AAV-GFP–treated mice began water restriction at statistically similar baseline body weights (Fig. 5*B*; *t*(15) = –1.456, *p* = 0.165). Importantly, all mice were maintained at statistically similar body weights throughout go/no-go task performance (Fig. 5*C*, *F*; *t*(15) = –0.043, *p* = 0.966). Further, we measured the amount of water provided to the mice after behavioral testing each day to maintain appropriate body weights (as shown in Fig. 5*C*). Each day, the experimenter provided supplemental water to the water-restricted mice (in addition to what they received upon making correct decisions). AAV-Cre–injected mice required more water than AAV-GFP–injected mice to maintain 80–85% body weight levels throughout the duration of experimentation (Fig. 5*D*; *t*(15) = 3.913, *p* = 0.0014). Pilot experiments (not included in Fig. 1) in which we provided AAV-Cre–injected mice with the same levels of water as AAV-GFP–injected mice revealed excessive weight loss in AAV-Cre–injected mice (data not shown), and therefore we established the paradigm of providing AAV-Cre–treated mice with additional supplemental water each day to ensure that mice in both treatment groups fell within a similar operational definition for thirst.

Finally, on a separate day (see Fig. 1), mice were placed in the operant chambers for a test of water motivation lasting 1 h. In this task, each nose poke into the odor port (in absence of odor) immediately triggered a reward to be released in the reward port, and, thus, this task allowed free access to water and quantification of water motivation. AAV-Cre–treated mice completed trial blocks with similar latencies (Fig. 5*Ei*; *t*(13) = –1.762, *p* = 0.102) and completed a similar number of blocks (Fig. 5*Eii*; *t*(13) = 1.76, *p* = 0.102) compared to AAV-GFP-treated mice. Together, these data from the above control measures suggests that “non-olfactory” behaviors of *Tph2*-targeted mice during engagement in the go/no-go test are not confounded by differences in the ability of these mice to execute coordinated motor behaviors or by altered levels of water motivation.

### Preserved higher-order odor-guided behaviors in *Tph2*-targeted mice

We next sought to explore the ability of *Tph2*-targeted mice to engage in higher-order olfactory-based behaviors. Reversal learning is a cognitive ability that is often explored in the context of learning and memory studies. Being reliant on the basic function of odor detection and identification, odor reversal learning, wherein the behavioral contingencies between the CS+ and CS– odors are switched so that the previously unrewarded odor is rewarded and *vice versa*, is a commonly used paradigm (Macrides et al., 1982; Schoenbaum et al., 1999; Sokolic and McGregor, 2007). 5-HT is considered in some contexts to be essential for odor learning (McLean et al., 1993) and cognitive flexibility (Lapiz-Bluhm et al., 2009; Coccaro et al., 2011). Thus, while not specifically assaying olfactory perception, we next investigated reversal learning to explore whether this function of olfactory sensory-dependent cognitive flexibility is impaired in 5-HT–depleted mice.

**Figure 6.**
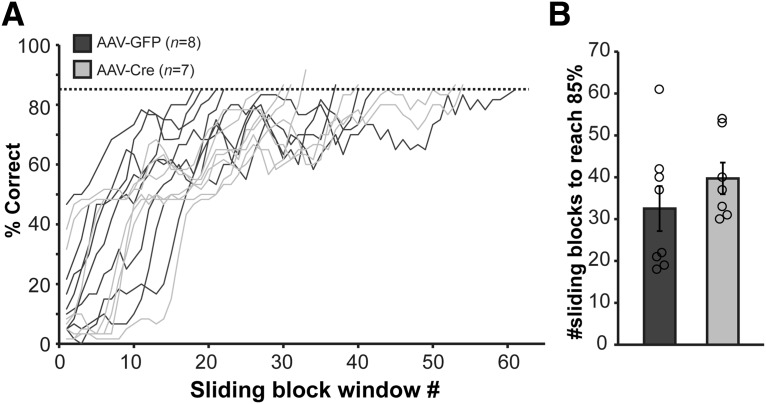
Adult brain 5-HT synthesis is not required for olfactory reversal learning. ***A***, Learning curves during odor-pair reversal, plotted with a three-block average sliding window until each mouse reached or surpassed 85% correct responses. ***B***, Average number of sliding blocks to reach ≥85% correct. Circles: values for individual mice.

We reversed the CS+ and CS– behavioral contingencies and monitored the responses of both AAV-GFP (*n* = 8) and AAV-Cre mice (*n* = 7) over successive daily sessions (Fig. 6*A*; all mice from cohorts 2 and 3). As was the case for the initial learning of the go/no-go task (Fig. 4*A–D*), both groups of mice displayed similar learning curves to acquire the reversal (Fig. 6*A*), with interanimal variability present in both groups. To reduce bin noise, learning curves were plotted and analyzed from sliding block window averages over three successive blocks. AAV-GFP– and AAV-Cre–injected mice required a similar number of sliding blocks to acquire the reversal to criterion level (Fig. 6*B*; *t*(13) = 1.067, *p* = 0.305). Thus, at least in this context, mice maintain cognitive flexibility in the absence of adult brain 5-HT.

### Confirmation of 5-HT synthesis deficiency in mice with perseverant olfactory behaviors and evidence that residual brain 5-HT does not contribute to go/no-go behavior

**Figure 7.**
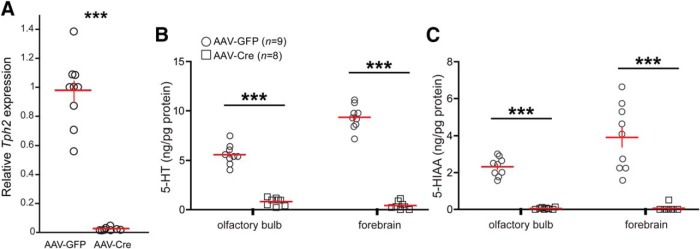
Confirmation of *Tph2* targeting and 5-HT depletion in cohorts used for behavior experiments. ***A***, qPCR results displaying a significant reduction in *Tph2* expression, relative to *Actb*, in the DRN region of *Tph2^fl/fl^* mice injected with AAV-Cre (*n* = 8) compared with untreated controls (*n* = 9). ***p < 0.0001. Data are from mice in cohorts 2 and 3. HPLC-quantified levels of 5-HT (***B***) and 5-HIAA (***C***) in the olfactory bulbs and forebrain of the same mice used for go/no-go behavior (cohorts 2 and 3). Tissue were collected immediately after the completion of the last behavioral measure (water motivation, Fig. 5*E*). Data are mean ± SEM. Individual points: individual mice. ***p < 0.0001.

We next verified loss of *Tph2* expression in the DRN of AAV-Cre–injected *Tph2^fl/fl^* mice used for the go/no-go testing (cohorts 2 and 3, *n* = 9 AAV-GFP and *n* = 8 AAV-Cre). Analysis of these mice demonstrated that this targeting approach resulted in a 98.01% decrease in *Tph2* mRNA in the DRN (*t*(15) = 11.254, *p* < 0.0001, AAV-Cre vs. AAV-GFP mice; Fig. 7*A*). Further, the brain of each mouse was collected to confirm the depletion of 5-HT (*n* = 9 AAV-GFP and *n* = 8 AAV-Cre). HPLC analysis confirmed robust depletions of 5-HT and in its main metabolite, 5-HIAA, in both olfactory bulb (5-HT, *t*(15) = 12.588, *p* < 0.0001; 5-HIAA, *t*(15) = 12.519, *p* < 0.0001) and forebrain (5-HT, *t*(15) = 19.847, *p* < 0.0001; 5-HIAA, *t*(15) = 6.043, *p* < 0.0001; Fig. 7*B*, *C*).

As we have presented (Figs. 2 and 7), this approach yielded near-complete eliminations of forebrain and olfactory bulb 5-HT. Whereas highly significant reductions in 5-HT were present in AAV-Cre–injected mice compared with AAV GFP mice across all animals in the olfactory bulb and forebrain, some 5-HT remained. Does the residual 5-HT—that is, the minor amount found in the olfactory bulbs or forebrain after AAV-Cre injection—contribute to odor discrimination as assayed here? To test this, we compared, within AAV-Cre–injected animals, the HPLC-determined concentration of residual 5-HT in both olfactory bulb and forebrain homogenates (same data as in Fig. 7*B*) to that of each animal’s percentage of correct responses [computed across 2000 trials (same data as in Fig. 4*E*)]. Again, supporting the notion that the olfactory system does not need 5-HT for its basic function, no relationship was observed between residual 5-HT in either the olfactory bulb (Pearson’s *r* = 0.321, *p* = 0.483, *n* = 7) or forebrain (Pearson’s *r* = 0.0964, *p* = 0.837, *n* = 7) and the percentage of correct responses.

## Discussion

In the present study, we sought to address the longstanding question: Do mammals need 5-HT for olfaction? More specifically, without adult brain 5-HT, do animals possess deficits in the most basic elements of olfactory-guided behaviors? The olfactory system is innervated by numerous neuromodulatory systems. These systems, including dopamine, acetylcholine, and, as explored herein, 5-HT, are hypothesized to provide critical refinements to the function of the olfactory system within the olfactory bulb, as well as in secondary and tertiary olfactory-processing stages (Linster and Fontanini, 2014). For instance, multiple lines of elegant work have revealed that cholinergic modulation within the olfactory bulb and downstream piriform cortex impacts fine odor discrimination, short-term odor memory, odor-based rule learning, and odor habituation (Ravel et al., 1992; Saar et al., 2001; Fletcher and Wilson, 2002; Linster and Cleland, 2002; Mandairon et al., 2006; Chaudhury et al., 2009). Despite intense interest in understanding serotonergic modulation of olfactory sensory input, surprisingly few studies have addressed the necessity of 5-HT in olfactory-guided behaviors in the intact animal. In one study, the serotonergic neurotoxin 5,7-dihydroxytrypmaine (5,7-DHT) was used to destroy 5-HT axonal inputs to the rat olfactory bulb (Moriizumi et al., 1994). The authors reported that 5,7-DHT–treated rats, trained to avoid cycloheximide in drinking water, became anosmic after neurotoxin treatment (Moriizumi et al., 1994). However, the behavioral assay was not specific for olfaction, and the reported behavioral impairment did not occur until several weeks after depletion of 5-HT immunoreactivity in the bulb. Moreover, the timing of anosmia was correlated with glomerular atrophy that also occurred a few weeks after 5,7-DHT treatment, suggesting that the behavioral deficits were not the result of 5-HT deficiency but rather long-term toxic effects of 5,7-DHT on olfactory structures. A different group reported that 5,7-DHT–based denervation of centrifugal raphe input to the rat neonatal olfactory bulb is required for odor learning and memory and concluded a functional role of 5-HT in this context (McLean et al., 1993). However, in *Lmx1b*-deficient mice, in which 5-HT neurons fail to differentiate and 5-HT synthesis is genetically blocked permanently at embryonic day 10.5, olfactory function remains intact (Liu et al., 2011b). The selective serotonin-reuptake inhibitor, fluoxetine, rescued deficits in olfactory acuity brought about in mice chronically treated with corticosterone but impaired olfaction when chronically administered to corticosterone-free mice (Siopi et al., 2016). Thus, the significance of 5-HT itself, specifically in the adult brain for odor-guided behaviors and also olfactory perception, is unclear. This void in part comes from a lack of methods to specifically target adult brain 5-HT synthesis.

In this study, we used a sensitive olfactory go/no-go test and conditionally targeted *Tph2* with AAV-Cre injections in the adult brain to specifically block 5-HT synthesis, thus avoiding potential off-target effects of pharmacological approaches. The olfactory go/no-go test is a sensitive assay of olfactory function, and qualitatively, mice in our task learned and performed within ranges reported by other groups (Bodyak and Slotnick, 1999; Kelliher et al., 2003; Abraham et al., 2004; Lin et al., 2004; Pho et al., 2005; Wesson et al., 2006). Although the targeting of *Tph2* caused a nearly complete elimination of 5-HT synthesis, this deficiency does not result in loss of 5-HT neuron cell bodies, as evidenced by their intact expression of aromatic amino acid decarboxylase (Whitney et al., 2016). The conditional genetic approach left intact early 5-HT signaling and thus did not interfere with 5-HT’s effects on sensory system organization and development (Cases et al., 1996; Gaspar et al., 2003; Toda et al., 2013). Further, this approach avoids potential but presently unknown compensatory mechanisms (Whitney et al., 2016) that might occur in response to 5-HT deficiency in the developing nervous system. The blockade of adult 5-HT synthesis did result in a significant and robust phenotype of altered water motivation (Fig. 5) and has been shown to impact activity levels and circadian rhythms (Whitney et al., 2016). However, in view of the profound effects of serotonergic function on olfactory circuitry, the present results were unexpected, as they indicate that adult brain 5-HT is not necessary for odor learning, coarse odor discrimination, or normal coordinated odor-guided behavior (sampling durations, Fig. 4*F*). Further, we found that *Tph2*-targeted mice were able to successfully complete a reversal learning task (Fig. 6). This finding was also surprising, as a significant number of studies using a range of approaches to alter 5-HT function and then assess behavioral outcomes in rodents, nonhuman primates, and humans have repeatedly suggested that reduced brain 5-HT levels hinder reversal learning (Murphy et al., 2002; Masaki et al., 2006; Bari and Robbins, 2013; Izquierdo et al., 2016). A potential explanation for our discordant findings is that the rat, not the mouse, has been the typical rodent species used to probe 5-HT’s role in reversal learning. In addition, because perturbation of different 5-HT receptor subtypes, which presumably influence distinct circuitry, produces opposing effects on reversal learning (Furr et al., 2012; Nilsson et al., 2012), perhaps a near-complete absence of forebrain 5-HT has a net counterbalancing effect on reversal learning. A similar explanation may account for the lack of effect of adult brain Tph2 targeting on olfaction. With respect to reversal learning outcomes, however, this explanation does not account for the discrepancy between our findings and earlier ones in which 5-HT was also broadly depleted from the forebrain and therefore would have been expected to impact 5-HT signaling through more than one 5-HT receptor subtype. A conspicuous difference between our study and earlier ones, however, is the method used to deplete brain 5-HT. In contrast to our viral/genetic approach, previous studies have used pharmacological or dietary means to reduce brain 5-HT (Clarke et al., 2004, 2005,2007; Masaki et al., 2006; Lapiz-Bluhm et al., 2009; Bari et al., 2010; Izquierdo et al., 2012, 2016). Although off-target pharmacological effects are not potentially inherent in our *Tph2* targeting approach, we cannot rule out the possibility that the severe deficiency of brain 5-HT in *Tph2* conditional knockout mice lasting several weeks before behavioral testing may have triggered presently unknown secondary effects, such as homeostatic alterations in the function of other transmitter systems, that compensate for the absence of 5-HT modulation of olfactory synaptic circuitry. Further parallel investigations of cognitive flexibility with adult *Tph2*-targeted mice and traditional methods of 5-HT depletion are needed to resolve the mechanistic basis of previously reported effects, which, our results suggest, are not directly due to altered 5-HT levels.

Our findings prompt the question as to what role 5-HT does play in olfaction. Why do the essential olfactory structures receive massive 5-HT innervation if not for even adjusting the most essential functioning of the system? One possibility is that 5-HT is needed only for the more nuanced aspects of odor-guided behavior, including the discrimination of structurally similar odorants (Cleland et al., 2002; Uchida and Mainen, 2003; Abraham et al., 2004), figure-background segregation (Barnes et al., 2008; Chapuis and Wilson, 2011), or intensity perception (Wojcik and Sirotin, 2014). For instance, a mouse may need 5-HT to discern the difference of odor in a sexually receptive female mouse versus an unreceptive one, yet may not need 5-HT to recognize the scent difference between a mouse and a predator. It is also possible that the influence of 5-HT would manifest only in cases of changes in behavioral state, including startle, arousal, or hunger. Nevertheless, our results, that the essential functions of the mammalian olfactory system can persevere despite a near-complete absence of adult brain 5-HT, are striking and suggest need for a refinement of models wherein 5-HT serves a major role in the olfactory system. Instead, we may benefit from recognizing a far more limited role of 5-HT in olfactory system function and perception. Further, this work provides a foundation to probe for compensatory synaptic mechanisms whereby our olfactory system may engage in informing behaviors, including those involving transmitter corelease (Trudeau, 2004; Zhou et al., 2005; Barker et al., 2016).

## Synthesis

The decision was a result of the Reviewing Editor Cheryl Sisk and the peer reviewers coming together and discussing their recommendations until a consensus was reached. A fact-based synthesis statement explaining their decision and outlining what is needed to prepare a revision is listed below. The following reviewers agreed to reveal their identity: Patricia Gaspar, Andrew Dacks

Two experts have reviewed your manuscript, and I concur with their evaluations. Both agree that the research is technically sound and has yielded results that will be of interest to the eNeuro readership. However, they point out that your findings of no effect of serotonin depletion on olfaction may be controversial, given the rich innervation of olfactory structures by serotonergic fibers, yet on the other hand, may not come as a surprise, because neuromodulators of sensory processing (serotonin, in this case) may exert subtle effects that are not revealed in the particular behavioral probes that you used. The reviewers also raise a number of alternative interpretations for you to consider, as well as some suggestions for strengthening/improving data presentation and analysis. In your revised manuscript, please address the reviewers' comments below.

Overall evaluation

1. The results demonstrate quite clearly that complete of serotonin depletion in adulthood is not required for primary olfactory discrimination and olfactory based operant learning. The model is convincing, the controls and analyses support the main conclusions. Despite these negative observations it is not possible, to entirely rule out a role of 5-HT in modulating olfactory processing. As acknowledged in the last paragraph of their discussion, serotonin may be needed for other olfactory-based behaviors, in a more physiological contexts. This is actually frequently the case for neuromodulators that are not required for primary sensory processing but are important to modulate more subtle and context-dependent sensory processing. My suggestion to the authors would therefore be to tone down their statements regarding the absence of any role of 5-HT in olfactory processing. Such a nuance could introduced in the abstract/introduction. It is also possible that effects of 5-HT depletion could appear only after a chronic depletion, in this regard the authors should discuss the paper of Morizuni et al. Neuroscience 1994, who showed long term changes in the OB after 5-7 DHT depletion in rats.

2. The manuscript seeks to determine the necessity of serotonin for performance of basic olfactory processing as measured via a Go/No-Go behavioral assay. The authors demonstrate very convincingly that adult brain serotonin has been eliminated and that mice in which serotonin production is eliminated perform, for the most part, identical to control mice in their assays. However, the claim that serotonin has little effect on olfaction will be controversial, as all olfactory structures in the brain are densely innervated by serotonergic processes and serotonin (whether exogenously or endogenously applied) has some fairly dramatic effects on the biophysical and synaptic properties of olfactory bulb neurons. Furthermore, several other studies have found behavioral effects of manipulating the serotonergic system, which the authors do not address in much depth. I therefore believe that the authors need to clarify certain points and emphasize other points in their paper to address this likely controversy.

Major Concerns

1. The authors state several times that they are testing basic olfactory capabilities and that serotonin likely plays a role in more nuanced olfactory processing. This is a very important point to emphasize and I believe that the authors do so to an adequate degree throughout the text. It would be strange to suggest that serotonin has no role in olfactory processing as the nervous system invests heavily in the targeting of serotonergic processes and the expression of serotonin receptors in the olfactory system. In addition, the myriad of recent studies demonstrating that serotonin affects physiological responses begs the question of why natural selection would cause the brain to invest so heavily in something of no use. I think that changing the title to "Preservation of basic olfactory processing in the absence of adult brain serotonin" more accurately reflects the main findings and should avoid readers dismissing this work as being contrary to previous work. It could also be changed to "Basic olfactory processing is preserved in the absence of adult brain serotonin" to avoid the passive voice.

2. An obvious objection to the results could be that perhaps the Go/No-Go task is not sensitive enough to detect the effects of such a manipulation. In other words, what if the task is so easy that the variability inherent to behavioral studies masks any effect that is present? Can the authors provide any evidence that this task is sensitive enough in their hands to behavioral deficits that could be caused by the general type of manipulation they've performed? Previous studies of their own or others using an identical set up would do and I would be surprised if they didn't exist. Also do the authors observe roughly the same degree of variability as observed by other groups using this task? Finally, could the authors please provide information as to the similarity of glomerular activation patterns for these odors and ease with which these odors are discriminated. Overtly making these counterarguments will allay reader doubts.

3. In the abstract, the authors state that they tested odor detection and found no effect. However, the detection thresholds were not tested with these mice. This is actually a task in which their manipulation could very likely have an effect (i.e. the sensitivity of the mice to low concentrations of odors) and should be added to list of tasks in which serotonin may play a role that the authors include at the end of the manuscript (such as fine odor discrimination). If the authors meant detection to mean the ability of the olfactory system to detect any concentration of odor, I don't know of any suggestions that 5-HT mediates olfactory responses, rather only that it modulates them.

4. There have been a variety of studies suggesting that serotonin production by the Raphe and the activity of Raphe neurons varies with time of day, peaking when animals are most active. This would include the work in the late 70s and early 80s by Jacobs and microdialysis measures from targets of the Raphe including the hippocampus. Because there is a circadian context to the effects of 5-HT is there any possibility that the reason that no effect was observed was due to the mice being tested during the light phase of their daily cycle? Could serotonin not normally have an effect at this time of day? I.e. could the animals not be getting tested within the context in which 5-HT is normally being released? The authors point out that their manipulation has been shown to affect circadian behavior, although the paper in question is In Press (and not accessible yet), so I can't evaluate how the observations of circadian deficits relate to the findings of this study.

5. The authors need to provide a more in-depth discussion of the discrepancy between their results and previous studies in which the behavioral role of 5-HT has been studied. One explanation provided is that the majority of the work has been done in rats, not mice. However, in the sentence before they state that 5-HT affects reversal learning in non-human primates and humans in addition to rodents, which would suggest that mice are the exception. In addition, the authors suggest that the one receptor subtype may oppose another receptor type lading to a net lack of effect. Couldn't the same argument of "no net effect" be made for eliminating serotonin in both non-olfactory brain regions vs olfactory brain regions? I.e. maybe the effects of 5-HT in the olfactory system are offset by loss of 5-HT in non-olfactory regions. Finally, a third explanation provided is that pharmacological or dietary means were used to reduce 5-HT as opposed to viral/genetic methods. The implication here is that the approaches used in previous studies had off-target effects. This is a slippery slope, as the authors have not demonstrated that their own approach lacks off-target effects. If a lack of off-target effects is demonstrated in Whitney et al In Press, then the authors should state this in the discussion.

6. In many cases, the authors use ANOVAs with a low power post-hoc test to analyze data sets comprised of two independent groups of animals. There were no normality tests performed and the appropriate test would have been either a student's unpaired T-test (if normally distributed) or a Mann-Whitney U test (if not normally distributed).

Minor concerns

1. In Figure 2 the serotonin-ir and Tph2-ir in the DRN and MRN are shown in red which is low luminance and sometimes difficult for red-green color blind people to interpret. These images should be changed to black and white.

2. I recognize that the order of validation data (figures 2, 5 and 7) was selected to follow the narrative of when the data was collected from each animal, i.e. cohort 1 was sacked purely to demonstrate via ICC that 5-HT and Tph2-ir are lost in these animals, whereas further cohorts were first behaviorally tested and then the effectiveness of the manipulation validated. Presenting the initial validations before the critical behavioral analyses could make the narrative of the study flow in a more cohesive manner.

3. Page 3 : reference to olfactory system of insects did not seem relevant here.

4. Page 3: reference to layer specific inputs the authors could add in their citation Muzerelle et al. 2016 showing similar laminar specificity of DR/MnR raphe inputs in mice.

5. page 14: Because there has been reported toxicity of Cre expression in some tissues, the authors should check with standard cellular staining that there are no lesions in the raphe.

6. page 15-line 332, wording is somewhat confusing: it is not the the targeting of Cre which is specific but indeed Cre which normally operates only on loxp sites.

## References

[B1] Abraham NM, Spors H, Carleton A, Margrie TW, Kuner T, Schaefer AT (2004) Maintaining accuracy at the expense of speed: stimulus similarity defines odor discrimination time in mice. Neuron 44:865–876. 10.1016/S0896-6273(04)00753-6 15572116

[B2] Aungst JL, Shipley MT (2005) Serotonin modulation of external tufted cells in mouse olfactory bulb glomeruli. Chem Senses 30:A146.

[B3] Bari A, Robbins TW (2013) Inhibition and impulsivity: behavioral and neural basis of response control. Prog Neurobiol 108:44–79. 23856628 10.1016/j.pneurobio.2013.06.005

[B4] Bari A, Theobald DE, Caprioli D, Mar AC, Aidoo-Micah A, Dalley JW, Robbins TW (2010) Serotonin modulates sensitivity to reward and negative feedback in a probabilistic reversal learning task in rats. Neuropsychopharmacology 35:1290–1301. 10.1038/npp.2009.233 20107431 PMC3055347

[B5] Barker DJ, Root DH, Zhang S, Morales M (2016) Multiplexed neurochemical signaling by neurons of the ventral tegmental area. J Chem Neuroanat 73:33–42. 10.1016/j.jchemneu.2015.12.016 26763116 PMC4818729

[B6] Barnes DC, Hofacer RD, Zaman AR, Rennaker RL, Wilson DA (2008) Olfactory perceptual stability and discrimination. Nat Neurosci 11:1378–1380. 10.1038/nn.2217 18978781 PMC2682180

[B7] Bodyak N, Slotnick B (1999) Performance of mice in an automated olfactometer: odor detection, discrimination and odor memory. Chem Senses 24:637–645. 10587496 10.1093/chemse/24.6.637

[B8] Bouret S, Sara SJ (2005) Network reset: a simplified overarching theory of locus coeruleus noradrenaline function. Trends Neurosci 28:574–582. 10.1016/j.tins.2005.09.002 16165227

[B9] Bowker RM, Westlund KN, Coulter JD (1981) Origins of serotonergic projections to the spinal cord in rat: an immunocytochemical-retrograde transport study. Brain Res 226:187–199. 10.1016/0006-8993(81)91092-1 7028211

[B10] Brill J, Shao Z, Puche AC, Wachowiak M, Shipley MT (2016) Serotonin increases synaptic activity in olfactory bulb glomeruli. J Neurophysiol 115:1208–1219. 10.1152/jn.00847.2015 26655822 PMC4808087

[B11] Brunert D, Tsuno Y, Rothermel M, Shipley MT, Wachowiak M (2016) Cell-type-specific modulation of sensory responses in olfactory bulb circuits by serotonergic projections from the raphe nuclei. J Neurosci 36:6820–6835. 10.1523/JNEUROSCI.3667-15.2016 27335411 PMC4916254

[B12] Cases O, Vitalis T, Seif I, De Maeyer E, Sotelo C, Gaspar P (1996) Lack of barrels in the somatosensory cortex of monoamine oxidase a–deficient mice: role of a serotonin excess during the critical period. Neuron 16:297–307. 10.1016/S0896-6273(00)80048-3 8789945

[B13] Chapuis J, Wilson DA (2011) Bidirectional plasticity of cortical pattern recognition and behavioral sensory acuity. Nat Neurosci 15:155–161. 10.1038/nn.2966 22101640 PMC3245808

[B14] Chaudhury D, Escanilla O, Linster C (2009) Bulbar acetylcholine enhances neural and perceptual odor discrimination. J Neurosci 29:52–60. 10.1523/JNEUROSCI.4036-08.2009 19129384 PMC2768367

[B15] Clarke HF, Dalley JW, Crofts HS, Robbins TW, Roberts AC (2004) Cognitive inflexibility after prefrontal serotonin depletion. Science 304:878 10.1126/science.1094987 15131308

[B16] Clarke HF, Walker SC, Crofts HS, Dalley JW, Robbins TW, Roberts AC (2005) Prefrontal serotonin depletion affects reversal learning but not attentional set shifting. J Neurosci 25:532–538. 10.1523/JNEUROSCI.3690-04.2005 15647499 PMC6725478

[B17] Clarke HF, Walker SC, Dalley JW, Robbins TW, Roberts AC (2007) Cognitive inflexibility after prefrontal serotonin depletion is behaviorally and neurochemically specific. Cereb Cortex 17:18. 10.1093/cercor/bhj120 16481566

[B18] Cleland TA, Morse A, Yue EL, Linster C (2002) Behavioral models of odor similarity. Behav Neurosci 116:222–231. 11996308 10.1037//0735-7044.116.2.222

[B19] Coccaro EF, Sripada CS, Yanowitch RN, Phan KL (2011) Corticolimbic function in impulsive aggressive behavior. Biol Psychiatry 69:1153–1159. 10.1016/j.biopsych.2011.02.032 21531387

[B20] Fletcher ML, Wilson DA (2002) Experience modifies olfactory acuity: acetylcholine-dependent learning decreases behavioral generalization between similar odorants. J Neurosci 22:RC201. 11784813 10.1523/JNEUROSCI.22-02-j0005.2002 PMC2365514

[B21] Furr A, Lapiz-Bluhm MD, Morilak DA (2012) 5-HT2A receptors in the orbitofrontal cortex facilitate reversal learning and contribute to the beneficial cognitive effects of chronic citalopram treatment in rats. Int J Neuropsychopharmacol 15:1295–1305. 10.1017/S1461145711001441 22008191 PMC3454536

[B22] Gaspar P, Cases O, Maroteaux L (2003) The developmental role of serotonin: news from mouse molecular genetics. Nat Rev Neurosci 4:1002–1012. 10.1038/nrn1256 14618156

[B23] Hardy A, Palouzier-Paulignan B, Duchamp A, Royet JP, Duchamp-Viret P (2005) 5-Hydroxytryptamine action in the rat olfactory bulb: in vitro electrophysiological patch-clamp recordings of juxtaglomerular and mitral cells. Neuroscience 131:717–731. 10.1016/j.neuroscience.2004.10.034 15730876

[B24] Hen R (1992) Of mice and flies: commonalities among 5-HT receptors. Trends Pharmacol Sci 13:160–165. 1589910 10.1016/0165-6147(92)90054-a

[B25] Hurley LM, Devilbiss DM, Waterhouse BD (2004) A matter of focus: monoaminergic modulation of stimulus coding in mammalian sensory networks. Curr Opin Neurobiol 14:488–495. 10.1016/j.conb.2004.06.007 15321070

[B26] Izquierdo A, Brigman JL, Radke AK, Rudebeck PH, Holmes A (2016) The neural basis of reversal learning: an updated perspective. Neuroscience. Advance online publication. doi:10.1016/j.neuroscience.2016.03.021. 10.1016/j.neuroscience.2016.03.021 PMC5018909 26979052

[B27] Izquierdo A, Carlos K, Ostrander S, Rodriguez D, McCall-Craddolph A, Yagnik G, Zhou F (2012) Impaired reward learning and intact motivation after serotonin depletion in rats. Behav Brain Res 233:494–499. 10.1016/j.bbr.2012.05.032 22652392 PMC3402622

[B28] Jacobs BL, Azmitia EC (1992) Structure and function of the brain serotonin system. Physiol Rev 72:165–229. 1731370 10.1152/physrev.1992.72.1.165

[B29] Johnson BA, Ho SL, Xu Z, Yihan JS, Yip S, Hingco EE, Leon M (2002) Functional mapping of the rat olfactory bulb using diverse odorants reveals modular responses to functional groups and hydrocarbon structural features. J Comp Neur 449:180–194. 10.1002/cne.10284 12115688

[B30] Kapoor V, Provost AC, Agarwal P, Murthy VN (2016) Activation of raphe nuclei triggers rapid and distinct effects on parallel olfactory bulb output channels. Nat Neurosci 19:271–282. 10.1038/nn.4219 26752161 PMC4948943

[B31] Katz PS (1999) Beyond neurotransmission: neuromodulation and its importance for information processing. Oxford University Press: Oxford, UK.

[B32] Kelliher KR, Ziesmann J, Munger SD, Reed RR, Zufall F (2003) Importance of the CNGA4 channel gene for odor discrimination and adaptation in behaving mice. Proc Natl Acad Sci U S A 100:4299–4304. 10.1073/pnas.0736071100 12649326 PMC153087

[B33] Kim J-Y, Kim A, Zhao Z-Q, Liu X-Y, Chen Z-F (2014) Postnatal maintenance of the 5-Ht1a-Pet1 autoregulatory loop by serotonin in the raphe nuclei of the brainstem. Mol Brain 7:1–11. 10.1186/1756-6606-7-48 24972638 PMC4086287

[B34] Lapiz-Bluhm MDS, Soto-Piña AE, Hensler JG, Morilak DA (2009) Chronic intermittent cold stress and serotonin depletion induce deficits of reversal learning in an attentional set-shifting test in rats. Psychopharmacology (Berl) 202:329–341. 10.1007/s00213-008-1224-6 18587666 PMC2634823

[B35] Lerch-Haner JK, Frierson D, Crawford LK, Beck SG, Deneris ES (2008) Serotonergic transcriptional programming determines maternal behavior and offspring survival. Nat Neurosci 11:1001–1003. 10.1038/nn.2176 19160496 PMC2679641

[B36] Lin W, Arellano J, Slotnick B, Restrepo D (2004) Odors detected by mice deficient in cyclic nucleotide-gated channel subunit A2 stimulate the main olfactory system. J Neurosci 24:3703–3710. 10.1523/JNEUROSCI.0188-04.2004 15071119 PMC6729751

[B37] Linster C, Cleland TA (2002) Cholinergic modulation of sensory representations in the olfactory bulb. Neural Netw 15:709–717. 10.1016/S0893-6080(02)00061-8 12371521

[B38] Linster C, Cleland TA (2016) Neuromodulation of olfactory transformations. Curr Opin Neurobiol 40:170–177. 10.1016/j.conb.2016.07.006 27564660

[B39] Linster C, Fontanini A (2014) Functional neuromodulation of chemosensation in vertebrates. Curr Opin Neurobiol 29:82–87. 10.1016/j.conb.2014.05.010 24971592 PMC4268319

[B40] Liu S, Aungst JL, Puche AC, Shipley MT (2011a) Serotonin modulates the population activity profile of olfactory bulb external tufted cells. J Neurophysiol 107:473–483. 10.1152/jn.00741.2011 22013233 PMC3349690

[B41] Liu Y, Jiang Y, Si Y, Kim J-Y, Chen Z-F, Rao Y (2011b) Molecular regulation of sexual preference revealed by genetic studies of 5-HT in the brains of male mice. Nature 472:95–99. 10.1038/nature09822 21441904 PMC4094133

[B42] Liu Z, Zhou J, Li Y, Hu F, Lu Y, Ma M, Feng Q, Zhang J, Wang D, Zeng J, Bao J, Kim J-Y, Chen Z-F, El Mestikawy S, Luo M (2014) Dorsal raphe neurons signal reward through 5-HT and glutamate. Neuron 81:1360–1374. 10.1016/j.neuron.2014.02.010 24656254 PMC4411946

[B43] Lottem E, Lörincz ML, Mainen ZF (2016) Optogenetic activation of dorsal raphe serotonin neurons rapidly inhibits spontaneous but not odor-evoked activity in olfactory cortex. J Neurosci 36:7–18. 10.1523/JNEUROSCI.3008-15.2016 26740645 PMC6601795

[B44] Macrides F, Eichenbaum HB, Forbes WB (1982) Temporal relationship between sniffing and the limbic theta rhythm during odor discrimination reversal learning. J Neurosci 2:1705–1711. 7143047 10.1523/JNEUROSCI.02-12-01705.1982 PMC6564372

[B45] Mandairon N, Ferretti CJ, Stack CM, Rubin DB, Cleland TA, Linster C (2006) Cholinergic modulation in the olfactory bulb influences spontaneous olfactory discrimination in adult rats. Eur J Neurosci 24:3234–3244 10.1111/j.1460-9568.2006.05212.x 17156384

[B46] Masaki D, Yokoyama C, Kinoshita S, Tsuchida H, Nakatomi Y, Yoshimoto K, Fukui K (2006) Relationship between limbic and cortical 5-HT neurotransmission and acquisition and reversal learning in a go/no-go task in rats. Psychopharmacology (Berl) 189:249–258. 10.1007/s00213-006-0559-0 17016708

[B47] McLean JH, Darby-King A, Sullivan RM, King SR (1993) Serotonergic influence on olfactory learning in the neonate rat. Behav Neural Biol 60:152–162. 10.1016/0163-1047(93)90257-I 7906939

[B48] McLean JH, Shipley MT (1987) Serotonergic afferents to the rat olfactory bulb: I. Origins and laminar specificity of serotonergic inputs in the adult rat. J Neurosci 7:3016–3028. 2822862 10.1523/JNEUROSCI.07-10-03016.1987 PMC6569188

[B49] Moriizumi T, Tsukatani T, Sakashita H, Miwa T (1994) Olfactory disturbance induced by deafferentation of serotonergic fibers in the olfactory bulb. Neuroscience 61 733–738. 7838372 10.1016/0306-4522(94)90396-4

[B50] Murphy F, Smith K, Cowen P, Robbins T, Sahakian B (2002) The effects of tryptophan depletion on cognitive and affective processing in healthy volunteers. Psychopharmacology (Berl) 163:42–53. 10.1007/s00213-002-1128-9 12185399

[B51] Muzerelle A, Scotto-Lomassese S, Bernard JF, Soiza-Reilly M, Gaspar P (2016) Conditional anterograde tracing reveals distinct targeting of individual serotonin cell groups (B5–B9) to the forebrain and brainstem. Brain Struct Funct 221:535–561. 10.1007/s00429-014-0924-4 25403254 PMC4750555

[B52] Nilsson SRO, Ripley TL, Somerville EM, Clifton PG (2012) Reduced activity at the 5-HT2C receptor enhances reversal learning by decreasing the influence of previously non-rewarded associations. Psychopharmacology (Berl) 224:241–254. 10.1007/s00213-012-2746-5 22644128

[B53] Paxinos G, Franklin K (2000) The mouse brain in stereotaxic coordinates, 2nd ed San Diego: Academic Press.

[B54] Petzold GC, Hagiwara A, Murthy VN (2009) Serotonergic modulation of odor input to the mammalian olfactory bulb. Nat Neurosci 12:784–791. 10.1038/nn.2335 19430472

[B55] Pfaffmann C, Goff WR, Bare JK (1958) An olfactometer for the rat. Science 128:1007–1008. 13592287 10.1126/science.128.3330.1007

[B56] Pho V, Butman ML, Cherry JA (2005) Type 4 phosphodiesterase inhibition impairs detection of low odor concentrations in mice. Behav Brain Res 161:245–253. 10.1016/j.bbr.2005.02.011 15922051

[B57] Ravel N, Vigouroux M, Elaagouby A, Gervais R (1992) Scopolamine impairs delayed matching in an olfactory task in rats. Psychopharmacol 109:439–443. 1365859 10.1007/BF02247720

[B58] Saar D, Grossman Y, Barkai E (2001) Long-lasting cholinergic modulation underlies rule learning in rats. J Neurosci 21:1385–1392. 11160410 10.1523/JNEUROSCI.21-04-01385.2001 PMC6762243

[B59] Schmidt LJ, Strowbridge BW (2014) Modulation of olfactory bulb network activity by serotonin: synchronous inhibition of mitral cells mediated by spatially localized GABAergic microcircuits. Learn Mem 21:406–416. 10.1101/lm.035659.114 25031366 PMC4105717

[B60] Schoenbaum G, Chiba AA, Gallagher M (1999) Neural encoding in orbitofrontal cortex and basolateral amygdala during olfactory discrimination learning. J Neurosci 19:1876–1884. 10024371 10.1523/JNEUROSCI.19-05-01876.1999 PMC6782178

[B61] Siopi E, Denizet M, Gabellec M-M, de Chaumont F, Olivo-Marin J-C, Guilloux J-P, Lledo P-M, Lazarini F (2016) Anxiety- and depression-like states lead to pronounced olfactory deficits and impaired adult neurogenesis in mice. J Neurosci 36:518–531. 10.1523/JNEUROSCI.2817-15.2016 26758842 PMC6602024

[B62] Skagerberg G, Björklund A (1985) Topographic principles in the spinal projections of serotonergic and non-serotonergic brainstem neurons in the rat. Neuroscience 15:445–480. 4022334 10.1016/0306-4522(85)90225-8

[B63] Slotnick B, Restrepo D (2001) Olfactometry with mice In: Current protocols in neuroscience. Wiley: New York. 10.1002/0471142301.ns0820s33 18428626

[B64] Smith RL, Baker H, Greer CA (1993) Immunohistochemical analyses of the human olfactory bulb. J Comp Neur 333:519–530. 7690371 10.1002/cne.903330405

[B65] Sokolic L, McGregor IS (2007) Benzodiazepines impair the acquisition and reversal of olfactory go/no-go discriminations in rats. Behav Neurosci 121:527–534. 10.1037/0735-7044.121.3.527 17592943

[B66] Steinfeld R, Herb JT, Sprengel R, Schaefer AT, Fukunaga I (2015) Divergent innervation of the olfactory bulb by distinct raphe nuclei. J Comp Neur 523:805–813. 10.1002/cne.23713 25420775 PMC4328392

[B67] Suzuki Y, Kiyokage E, Sohn J, Hioki H, Toida K (2015) Structural basis for serotonergic regulation of neural circuits in the mouse olfactory bulb. J Comp Neur 523:262–280. 10.1002/cne.23680 25234191

[B68] Toda T, Homma D, Tokuoka H, Hayakawa I, Sugimoto Y, Ichinose H, Kawasaki H (2013) Birth regulates the initiation of sensory map formation through serotonin signaling. Dev Cell 27:32–46. 10.1016/j.devcel.2013.09.002 24135230

[B69] Trudeau LE (2004) Glutamate co-transmission as an emerging concept in monoamine neuron function. J Psychiatry Neurosci 29:296–310. 15309046 PMC446224

[B70] Uchida N, Mainen ZF (2003) Speed and accuracy of olfactory discrimination in the rat. Nat Neurosci 6:1224–1229. 14566341 10.1038/nn1142

[B71] Walther DJ, Peter JU, Bashammakh S, Hörtnagl H, Voits M, Fink H, Bader M (2003) Synthesis of serotonin by a second tryptophan hydroxylase isoform. Science 299:76 10.1126/science.1078197 12511643

[B72] Wesson DW, Keller M, Douhard Q, Baum MJ, Bakker J (2006) Enhanced urinary odor discrimination in female aromatase knockout (ArKO) mice. Horm Behav 49:580–586. 10.1016/j.yhbeh.2005.12.013 16448653 PMC2263132

[B73] Whitney MS, Shemery A, Yaw A, Donovan L, Glass JD, Deneris ES (2016) Adult brain serotonin deficiency causes hyperactivity, circadian disruption, and elimination of siestas. J Neurosci 36:9828–9842. 10.1523/JNEUROSCI.1469-16.2016 27656022 PMC5030349

[B74] Wojcik PT, Sirotin YB (2014) Single scale for odor intensity in rat olfaction. Curr Biol 24:568–573. 10.1016/j.cub.2014.01.059 24560575

[B75] Zhou F-M, Liang Y, Salas R, Zhang L, De Biasi M, Dani JA (2005) Corelease of dopamine and serotonin from striatal dopamine terminals. Neuron 46:65–74. 10.1016/j.neuron.2005.02.010 15820694

